# Factors Associated with Reduced Clinical Response in Adult ADHD: The Role of Alcohol and Cannabis Use Disorders and Autism Spectrum Disorder

**DOI:** 10.3390/jcm15072688

**Published:** 2026-04-02

**Authors:** Manuel Glauco Carbone, Beniamino Tripodi, Irene Matarese, Alessandro Bellini, Roberta Rizzato, Claudia Tagliarini, Filippo Della Rocca, Francesco De Dominicis, Icro Maremmani, Giulio Perugi, Angelo G. I. Maremmani

**Affiliations:** 1 Division of Psychiatry, Department of Medicine and Surgery, University of Insubria, 21100 Varese, Italy; beniamino.tripodi90@gmail.com (B.T.); alessandro.bellini1992@gmail.com (A.B.); roberta-rizzato@libero.it (R.R.); 2 VP Dole Research Group, G. De Lisio Institute of Behavioural Sciences, 56127 Pisa, Italy; filippo.dellarocca@yahoo.it (F.D.R.); icromaremmani@gmail.com (I.M.); angelogiovanniicro.maremmani@unicamillus.org (A.G.I.M.); 3 Division of Psychiatry, Department of Mental Health and Addictions, ASST Crema, 26013 Crema, Italy; 4 Clinical and Health Psychology Unit, Department of Surgical, Medical, Molecular and Critical Area Pathology, University of Pisa, 56126 Pisa, Italy; irenematarese@gmail.com; 5 Department of Mental Health, Psychiatric Diagnosis and Treatment Service, Sant’Elia Hospital, ASP 2, 93100 Caltanissetta, Italy; claudiatagliarini8@gmail.com; 6 Addiction Unit, Department of Mental Health and Addictions, ASL 5 Liguria, 19124 La Spezia, Italy; 7 Division of Psychiatry, Department of Mental Health, U.S.L. Umbria 2, 06049 Spoleto, Italy; francesco.dedominicis@gmail.com; 8 Faculty of Medicine, UniCamillus—Saint Camillus International University of Health and Medical Sciences, 00131 Rome, Italy; 9 Section of Psychiatry, Department of Clinical and Experimental Medicine, University of Pisa, 56126 Pisa, Italy; giulio.perugi@gmail.com

**Keywords:** adult ADHD, substance use disorders, cannabis use disorder, alcohol use disorder, treatment response

## Abstract

**Background:** Attention-Deficit/Hyperactivity Disorder (ADHD) in adulthood is frequently associated with complex psychiatric comorbidity, including high rates of Substance Use Disorders (SUDs), which may influence treatment outcomes. Although pharmacological treatments are effective for core ADHD symptoms, real-world response remains heterogeneous, and the contribution of specific substance-related and neurodevelopmental factors to treatment response is not fully understood. **Methods:** This retrospective observational study examined a real-world cohort of 67 adults with ADHD treated pharmacologically in a specialized outpatient setting. ADHD was diagnosed according to DSM-5-TR criteria using the Diagnostic Interview for ADHD in Adults (DIVA-5). Autism spectrum disorder (ASD) was recorded based on documented pre-existing specialist diagnoses and confirmed clinically at baseline. Psychiatric comorbidities and substance use disorders, including alcohol and cannabis use disorders, were assessed according to DSM-5-TR criteria. Clinical response was defined using the Clinical Global Impression–Improvement scale (CGI-I; responders = scores 1–3). Exploratory binary logistic regression analyses were used to identify clinical factors associated with treatment response. Given the limited sample size, revised multivariable models were specified parsimoniously on the basis of a priori clinical relevance. **Results:** At follow-up, 48 of 67 patients (71.6%) met criteria for clinical response. In revised parsimonious multivariable models, alcohol use disorder (OR ≈ 0.08–0.10, *p* = 0.010–0.026) and cannabis use disorder (OR ≈ 0.20–0.24, *p* = 0.014–0.028) were consistently associated with reduced odds of clinical response. Autism spectrum disorder showed a descriptive trend toward lower response rates but did not retain statistical significance after adjustment (*p* ≈ 0.11–0.15). **Conclusions:** In adults with ADHD treated in routine clinical practice, alcohol and cannabis use disorders were associated with a reduced likelihood of achieving clinically meaningful improvement under routine pharmacological care, whereas autism spectrum disorder showed a trend toward lower response that was not stable enough to support firm conclusions. These findings should be considered exploratory given the retrospective design, limited sample size, and lack of systematic treatment exposure measures.

## 1. Introduction

Attention-Deficit/Hyperactivity Disorder (ADHD) is currently conceptualized as a lifelong neurodevelopmental condition, with a substantial proportion of affected individuals continuing to experience clinically significant symptoms into adulthood [1]. In adult populations, ADHD is rarely an isolated disorder and is instead frequently embedded within complex psychopathological profiles characterized by high rates of psychiatric comorbidity [2]. Mood disorders, anxiety disorders, personality disorders, and Substance Use Disorders (SUDs) are particularly prevalent and contribute to functional impairment, diagnostic delay, and variability in treatment outcomes [3]. As a result, the clinical management of adult ADHD often requires an integrated and individualized approach that goes beyond the treatment of core attentional symptoms alone [4].

Within this framework of clinical complexity, increasing attention has been directed toward the role of co-occurring neurodevelopmental conditions. Autism spectrum disorder (ASD), in particular, frequently co-occurs with ADHD across the lifespan and shares substantial genetic liability, partially overlapping neurocognitive profiles, and common alterations in executive functioning, reward processing, and emotional regulation [5,6,7,8]. In adulthood, the ADHD–ASD co-occurrence has been associated with greater functional impairment, increased psychiatric burden, and more complex clinical trajectories, factors that may potentially influence treatment response [9,10]. Despite growing recognition of this overlap, the impact of ASD on pharmacological treatment outcomes in adult ADHD remains insufficiently explored in routine clinical settings.

Among psychiatric comorbidities, SUDs represent one of the most frequent and clinically challenging conditions associated with adult ADHD [11]. Epidemiological and clinical studies consistently demonstrate a bidirectional relationship between ADHD and substance use, whereby ADHD confers an increased risk for the development of SUDs, while substance use may exacerbate attentional deficits, emotional dysregulation, and executive dysfunction [12,13]. This relationship is thought to reflect shared neurobiological vulnerabilities, including dysregulation of dopaminergic reward pathways, impaired inhibitory control, and altered emotional processing [14]. Consequently, individuals with ADHD and comorbid SUDs often present with greater clinical complexity and poorer functional outcomes compared to those without substance use [15].

Cannabis and alcohol use disorders are particularly common in adults with ADHD and deserve specific clinical attention [16,17]. Research indicates that adults with ADHD are approximately three times more likely to exhibit Cannabis Use Disorder (CUD). Accordingly, lifetime prevalence of CUD among individuals with ADHD is approximately 27%, while the current prevalence is around 19% [18]. Cannabis use has been associated with alterations in dopaminergic signaling, executive functioning, and motivational processes, domains that are already compromised in ADHD [19,20,21,22]. Chronic exposure to Δ9-tetrahydrocannabinol (THC) may further impair attentional control, working memory, and emotional regulation, potentially interfering with the mechanisms through which ADHD pharmacological treatments exert their clinical effects [23,24], although the clinical relevance of these mechanisms for treatment response remains uncertain.

Similarly, ADHD is considered a relevant risk factor for developing Alcohol Use Disorder (AUD), with an odds ratio of 1.5. A lifetime prevalence of any AUD up to 43% and a 3 to 11% prevalence of moderate to severe AUD has been reported in ADHD subjects, with higher rates in adults than in adolescents. The co-occurrence of ADHD and AUD has been shown to lead to earlier AUD onset, more severe ADHD symptoms, polydrug use, and poorer outcomes, including higher rates of relapse and hospitalization. Moreover, comparative longitudinal studies suggest that alcohol consumption may begin to escalate slightly faster in individuals with ADHD already in early adolescence compared with non-ADHD peers, supporting the hypothesis of an accelerated substance-use trajectory [25,26].

Impulsivity, a common feature of ADHD, can contribute to binge drinking and reduced treatment adherence in individuals with ADHD and AUD [27]. AUD is linked to dysfunction of prefrontal and fronto-striatal circuits, reward processing abnormalities, and deficits in emotional self-regulation [28,29,30]. In individuals with ADHD, these neurobiological effects may overlap with core pathophysiological mechanisms of the disorder, contributing to persistent symptoms and reduced adaptive capacity [31]; however, the extent to which these alterations translate into differences in pharmacological treatment response remains to be clarified.

Pharmacological treatments targeting ADHD, including stimulant and non-stimulant medications, have demonstrated efficacy in reducing core symptoms and improving overall functioning in adult patients [32,33]. However, treatment response is highly heterogeneous, particularly in clinically complex and comorbid populations, especially in cases where SUDs co-occur [34,35,36]. While previous research has extensively examined the efficacy and tolerability of ADHD medications, fewer studies have specifically addressed factors associated with variability in clinical response. In particular, the role of SUD has often been investigated in terms of treatment safety, misuse liability, or effects on substance-related outcomes, rather than as potential clinical correlates of variability in ADHD treatment response [37].

Moreover, most available evidence derives from randomized controlled trials conducted in highly selected samples, which frequently exclude patients with active or significant comorbid substance use [38,39]. As a result, the applicability of these findings to real-world clinical settings remains limited [40]. There is a growing need for observational studies based on routine clinical practice that reflect the complexity of patients encountered in everyday psychiatric care and allow for the identification of factors associated with differential treatment response in adulthood [41].

Within this framework, cannabis and alcohol use disorders may be conceptualized not merely as comorbid conditions, but as clinical markers potentially associated with a lower probability of achieving clinically observable improvement under routine treatment conditions, rather than as direct determinants of pharmacological resistance. Importantly, this perspective does not imply that individuals with ADHD and comorbid substance use are unable to benefit from treatment, but rather that substance-related factors may influence the likelihood and magnitude of clinical improvement. While the association between ADHD and SUD-related clinical complexity is well established, fewer real-world studies in adult populations have specifically examined whether distinct SUD subtypes are differentially associated with clinician-rated global improvement during routine ADHD pharmacological treatment. In particular, most available evidence derives from randomized controlled trials or focuses on symptom-specific outcomes, whereas less is known about factors associated with overall clinical improvement as assessed in routine care settings.

The aim of the present study was to explore, in an exploratory and hypothesis-generating framework, clinical and psychopathological correlates of clinician-rated global improvement in adults with ADHD treated pharmacologically in a real-world setting. Using a retrospective observational design based on routinely collected clinical data, we examined whether SUDs, neurodevelopmental characteristics, clinical features, psychiatric comorbidities, and familial vulnerability factors were associated with variability in treatment response, as assessed by global improvement measures, in adults with ADHD receiving standard pharmacological care.

## 2. Materials and Methods

### 2.1. Study Design

This study is a retrospective observational analysis conducted in a real-world clinical setting and based on routinely collected clinical data. The investigation was based on anonymized data routinely collected during standard psychiatric care and did not involve any experimental intervention, randomization, or modification of usual treatment practices. All clinical decisions, including pharmacological prescriptions, were made independently by treating clinicians according to current clinical guidelines and individual clinical judgment.

Clinical and psychometric assessments were routinely performed at baseline (T0) and during follow-up visits as part of standard care. Baseline assessment (T0) corresponded to the initial clinical evaluation and included comprehensive diagnostic procedures, psychometric testing, and initiation or optimization of pharmacological treatment for ADHD. A first follow-up assessment (T1) was typically conducted approximately 3 months after treatment initiation during routine outpatient visits, with the aim of monitoring symptom severity, functional status, and early treatment response. A final follow-up assessment (T2) corresponded to the end of the observational period and generally occurred within 6 months of treatment initiation, depending on individual clinical trajectories and attendance patterns.

For the purposes of the present analysis, only patients with complete clinical data available at baseline (T0) and at the final follow-up assessment (T2) were included. Data collected at T0 and at T2 were retrospectively extracted and analyzed to explore clinical factors associated with response to pharmacological treatment for adult ADHD. Given the retrospective design, no formal control over treatment exposure, dose optimization, or adherence was possible, and these variables could not be systematically quantified. Clinical response was defined on the basis of global clinical improvement assessed at the final follow-up evaluation (T2), reflecting routine clinical judgment rather than a standardized outcome measure.

This study was conducted in accordance with the principles of the Declaration of Helsinki. Given the retrospective observational design and the use of fully anonymized data collected during routine clinical care, formal ethical approval was not required according to applicable national regulations.

### 2.2. Sample Recruitment and Participants

The study sample was derived from a retrospective review of clinical records of adult patients (≥18 years) followed at specialized outpatient ADHD clinics within the Department of Mental Health and Addictions of ASST Crema and the Department of Mental Health, Division of Psychiatry, U.S.L. Umbria 2, Spoleto, Italy. The sample represents a convenience cohort of consecutively evaluated patients within routine clinical practice. Eligible cases were identified among patients who had received a formal diagnosis of ADHD in adulthood and had been treated pharmacologically as part of routine clinical care.

For inclusion in the present analysis, clinical records were required to document:

(i) a diagnosis of ADHD established according to DSM-5-TR criteria and confirmed through structured clinical assessment and a standardized diagnostic interview;
(ii) initiation or ongoing treatment with ADHD-specific pharmacotherapy during the observation period;
(iii) availability of a baseline clinical assessment and at least one follow-up evaluation allowing the assessment of clinical response.

No a priori sample size calculation was performed, given the retrospective nature of the study, and the sample size was determined by the availability of complete clinical data.

Clinical records were excluded from the analysis if they referred to patients with subthreshold ADHD symptoms without a formal diagnosis, severe or unstable medical conditions contraindicating psychopharmacological treatment, acute psychiatric conditions requiring immediate hospitalization or major treatment changes, or insufficient clinical information to reliably evaluate treatment response.

Patients receiving concomitant psychopharmacological treatments for comorbid psychiatric conditions were included in the analysis, provided that these treatments were already in place and clinically stable for at least two months prior to the initiation of ADHD-specific pharmacotherapy and remained unchanged throughout the observation period, as documented in the clinical records. However, given the observational design, residual confounding related to concomitant treatments cannot be excluded.

### 2.3. Clinical Assessment and Diagnostic Procedures

All patients underwent a comprehensive psychiatric evaluation as part of routine clinical care. The diagnosis of ADHD in adulthood was established through a structured clinical interview and confirmed using the Diagnostic Interview for ADHD in Adults, version 5 (DIVA-5), administered by experienced psychiatrists [42]. The DIVA-5 was used as the primary diagnostic tool to assess the presence of childhood-onset and current ADHD symptoms, functional impairment across multiple life domains, and symptom persistence according to DSM-5-TR criteria.

Psychiatric comorbidities were systematically assessed using the Structured Clinical Interview for DSM-5 (SCID-5) [43], allowing for the identification of mood disorders, anxiety disorders, personality disorders, substance use disorders, neurodevelopmental disorders, and other relevant psychiatric conditions. For the purposes of the present analyses, SUD variables reflected active diagnoses at baseline. Diagnostic assessments were conducted by trained clinicians with expertise in adult ADHD and comorbid psychopathology.

For the purposes of the present analysis, comorbid conditions were operationalized as categorical variables (presence/absence of diagnosis), based on clinically established DSM-5-TR diagnoses. While this approach reflects routine clinical decision-making, it does not capture the dimensional severity or longitudinal course of comorbid conditions, particularly in the case of substance use disorders and neurodevelopmental traits.

ASD was recorded when a pre-existing DSM-5-TR diagnosis from specialist services was documented in the clinical records and subsequently confirmed through clinical reassessment at baseline, including a detailed developmental history.

As part of standard clinical monitoring, patients underwent a multidimensional psychometric assessment at baseline and during follow-up visits. Core ADHD symptomatology was evaluated using the Adult ADHD Self-Report Scale (ASRS) [44] and the Conners’ Adult ADHD Rating Scale—Observer Report: Screening Version (CAARS-O:SV) [45]. The CAARS-O:SV provided hetero-evaluated measures of inattentive symptoms, hyperactive/impulsive symptoms, total ADHD symptom burden, and an overall ADHD index, allowing for a detailed characterization of symptom dimensions.

General psychopathology was assessed using the Brief Psychiatric Rating Scale (BPRS) [46]. In addition to the total BPRS score, a specific six-item subscale (BPRS-6) [47], reflecting psychotic-like and disorganization symptoms (conceptual disorganization, hallucinations, unusual thought content, blunted affect, motor retardation, and emotional withdrawal), was calculated to provide a more sensitive measure of severe psychopathological features.

Mood symptoms were evaluated using the Hamilton Depression Rating Scale (HAM-D) [48] for depressive symptomatology and the Young Mania Rating Scale (YMRS) [49] for manic or hypomanic symptoms. These scales were used to monitor affective symptom severity over time and to identify potential mood instability during ADHD pharmacological treatment.

Executive functioning in everyday life was assessed using the Behavior Rating Inventory of Executive Function—Adult Version (BRIEF-A) [50]. Analyses focused on the two main indices: the Behavioral Regulation Index (BRI), reflecting the ability to modulate behavior and emotional responses, and the Metacognition Index (MI), reflecting planning, organization, working memory, and task monitoring. The Global Executive Composite was not used in order to preserve the distinction between regulatory and metacognitive domains.

Emotional dysregulation was assessed using the Reactivity, Intensity, Polarity and Stability questionnaire (RIPoSt-40) [51], a validated self-report instrument designed to capture multiple dimensions of emotional reactivity and regulation. In the absence of established clinical cut-offs, emotional dysregulation severity was treated as a dimensional variable. For selected analyses, patients were categorized into higher and lower emotional dysregulation (HED/LED) groups using a median split approach, consistent with prior research in dimensional psychopathology. This categorization should be interpreted cautiously, as it represents a statistical simplification of a continuous construct.

Circadian rhythm characteristics were evaluated using the Morningness–Eveningness Questionnaire (MEQ) [52]. A delayed sleep phase profile (DSP) was defined according to established cut-off values, reflecting a marked eveningness tendency.

Global clinical improvement was assessed using the Clinical Global Impression—Improvement scale (CGI-I) [53], which served as the primary outcome measure for defining treatment response. For the main analyses, treatment response was operationalized dichotomously (responder vs. non-responder) based on CGI-I ratings, in line with common practice in observational clinical studies, although this approach may reduce sensitivity to gradations of clinical change, particularly in heterogeneous clinical populations.

In addition, no standardized measures of ASD severity were systematically available in the dataset, limiting the possibility of exploring dimensional effects of neurodevelopmental traits on treatment response.

### 2.4. Definition of Clinical Response

Clinical response was defined a priori based on CGI-I scores at follow-up. Patients were classified as responders if they achieved a CGI-I score of 1 (very much improved), 2 (much improved), or 3 (minimally improved). Patients with CGI-I scores ≥ 4 were classified as non-responders.

This categorical outcome was selected to reflect a clinically meaningful global improvement observable in real-world clinical practice, rather than isolated changes in single symptom dimensions, and to ensure methodological consistency with the retrospective observational design of the study. However, this approach may reduce sensitivity to gradations of clinical change and should be interpreted in light of the heterogeneity of the sample.

### 2.5. Pharmacological Treatment

Pharmacological treatments included stimulant and non-stimulant medications approved or commonly used for the treatment of adult ADHD (methylphenidate, atomoxetine, and bupropion). The choice of medication, dosing, and titration schedules was based on routine clinical practice and determined by the treating clinicians according to individual clinical characteristics and patient needs. These included ADHD symptom profile and severity, presence and type of psychiatric and neurodevelopmental comorbidities, substance use history, cardiovascular and medical risk factors, previous treatment response or tolerability, and patient preferences.

Dose adjustments were performed during follow-up visits as part of standard clinical care, with gradual titration aimed at achieving optimal symptom control while maintaining an acceptable tolerability profile.

Given the retrospective design, detailed and standardized data on medication dosage, treatment adherence, and cumulative drug exposure were not consistently available across all patients and were therefore not included as quantitative variables in the analyses. Consequently, the present findings should be interpreted with caution, as differences in treatment response may be partially influenced by variability in pharmacological exposure rather than solely by patient-related clinical characteristics.

### 2.6. Substance Use Variables

SUDs were identified through clinical assessment according to DSM-5-TR criteria [54]. In the present sample, cases coded as SUD reflected active substance use disorders at baseline (T0). Individuals with a past or remitted SUD who did not meet criteria for an active disorder at T0 were not classified as having SUD in the present analyses.

Consistent with the overall analytical strategy, SUD variables were operationalized dichotomously (presence/absence). This represents a clinically pragmatic approach reflecting routine diagnostic practice; however, it entails a substantial loss of granularity, as it does not capture severity, duration, patterns, or recency of substance use, and should be considered an important limitation of the study.

All SUD categories (including alcohol, cannabis, stimulant, and opioid use disorders) were initially entered into the regression analyses as separate dichotomous variables. Selection of variables for the revised multivariable models was based primarily on a priori clinical relevance, with univariate findings considered in a secondary exploratory manner, in order to reduce overfitting given the limited sample size. Cannabis use disorder (CUD) and alcohol use disorder (AUD) were retained in the final model, as they showed significant associations with clinical response in multivariable analyses.

### 2.7. Statistical Analysis

Statistical analyses were conducted to explore demographic, clinical, and psychopathological characteristics of the sample and to investigate factors associated with clinical response to ADHD pharmacological treatment.

Descriptive statistics were used to summarize sample characteristics. Continuous variables are summarized as means and standard deviations (SDs), while categorical variables are presented as frequencies and percentages.

Between-group comparisons (responders vs. non-responders) were performed using independent-samples *t*-tests for continuous variables and chi-square (χ^2^) or Fisher’s exact tests for categorical variables, as appropriate based on expected cell counts in contingency tables.

To examine factors associated with clinical response, exploratory binary logistic regression analyses were conducted to estimate associations between selected variables and clinical response. Univariate analyses were initially conducted to explore crude associations between individual variables and clinical response.

In the revised analyses, multivariable models were specified parsimoniously using a limited number of clinically relevant variables selected a priori, in order to reduce overfitting risk in the context of a limited sample size. Alcohol use disorder and cannabis use disorder were included based on the study hypothesis, while age, sex, stimulant treatment exposure, autism spectrum disorder, and family history of neurodevelopmental disorders were evaluated in sensitivity models.

Given the limited sample size, the number of variables included in each multivariable model was restricted in accordance with recommended events-per-variable criteria for logistic regression models. In addition, given the relatively low number of non-responders and the small number of exposed cases for some variables, regression coefficients and confidence intervals may be unstable and should be interpreted cautiously. Potential discrepancies between univariate and multivariable findings were interpreted as reflecting adjustment for confounding and intercorrelations among variables, but should not be considered, in themselves, evidence of stable adjusted associations. Rather, such findings were interpreted as exploratory adjusted associations requiring replication in larger samples.

Odds ratios (ORs) with 95% confidence intervals (CIs) are reported. Given the retrospective design and limited sample size, all multivariable findings should be interpreted as exploratory and hypothesis-generating in nature.

All statistical analyses were performed using IBM SPSS Statistics, version 23 [55].

### 2.8. Use of Generative AI

Generative AI tools (ChatGPT, OpenAI, GPT-4 architecture, accessed in March 2026) were used only for minor language editing and stylistic refinement of the English text. No AI tools were used for data generation, analysis, interpretation, or development of the scientific content.

## 3. Results

### 3.1. Sample Characteristics

The final analytic sample included 67 adults with ADHD (mean age 29.09 ± 10.20 years; 21 women, 31.3%), all with complete baseline and follow-up assessments. The main demographic and clinical characteristics of the sample are summarized in Table 1.

ADHD presentation was predominantly combined (n = 45, 67.2%), followed by inattentive (n = 17, 25.4%) and hyperactive/impulsive (n = 5, 7.5%).

Psychiatric family history was frequent, mainly for mood disorders (n = 34, 50.7%) and neurodevelopmental disorders (n = 19, 28.4%); family history for SUDs was reported in 4 patients (6.0%).

Comorbid psychiatric diagnoses were common in the sample. Mood disorders were present in 42 patients (62.7%), including one case with psychotic features, and anxiety disorders in 36 patients (53.7%). Personality disorders were diagnosed in 20 patients (29.9%), while eating disorders were less frequent (n = 3, 4.5%). SUDs were identified in 37 patients (55.2%). Within SUDs, CUD was the most prevalent (n = 21, 31.3%), followed by stimulant use disorder (n = 9, 13.4%), AUD (n = 6, 9.0%), and Opioid Use Disorder (OUD) (n = 1, 1.5%).

Neurodevelopmental comorbidities were also common. Specific learning disorders were diagnosed in 16 patients (23.9%), ASD in 7 patients (10.4%), tic disorders in 6 patients (9.0%), and intellectual disability in 6 patients (9.0%).

Regarding ADHD pharmacotherapy, methylphenidate (MPH) was prescribed in 45 patients (67.2%), atomoxetine (ATX) in 17 (25.4%), and bupropion in 5 (7.5%). Medication dosing was adjusted during routine clinical care; however, detailed and standardized data on dosage and cumulative exposure were not consistently available and are therefore not reported quantitatively.

Concomitant treatments for comorbid conditions were frequent (e.g., antidepressants 25.4%, mood stabilizers/antiepileptics 20.9%, antipsychotics 17.9%).

Overall, 48 patients (71.6%) met responder criteria at follow-up based on CGI-I, while 5 patients (7.5%) met criteria for remission (CGI-I = 1).

Adverse events were infrequent and descriptively recorded. Two cases of hypomanic switch were observed during treatment with MPH, and one case of behavioral worsening occurred in a patient with comorbid ADHD and ASD, which was managed through intensified clinical monitoring. The remaining patients reported predominantly mild and transient side effects.

### 3.2. Descriptive and Exploratory Analyses

Across the overall sample, psychometric measures showed descriptive improvement trends from baseline to follow-up across multiple clinical domains, including global psychopathology, mood symptoms, ADHD core symptoms, and emotional dysregulation. Given the exploratory nature of these analyses and the heterogeneity of available measures, these changes are reported descriptively and were not included in formal inferential testing. Baseline differences across ADHD presentation subtypes were observed in several domains but largely attenuated at follow-up.

Univariate analyses exploring associations between responder status (responders vs. non-responders) and categorical clinical variables showed no statistically significant associations across most variables. Most family history variables and current psychiatric comorbidities were not significantly associated with clinical response.

For substance use disorder categories, alcohol use disorder (AUD) was associated with response status in chi-square analysis (χ^2^ = 4.76, *p* = 0.029), although Fisher’s exact test yielded a borderline result (*p* = 0.050), reflecting low expected cell counts in contingency tables. Similarly, cannabis use disorder (CUD) showed a non-significant trend (χ^2^ = 3.17, *p* = 0.075; Fisher’s exact *p* = 0.088). Autism spectrum disorder (ASD) also showed a non-significant trend toward lower response rates (χ^2^ = 3.19, *p* = 0.074; Fisher’s exact *p* = 0.094).

Responder and non-responder distributions according to AUD, CUD, and ASD are reported in Table 2.

Given the small sample size and the presence of cells with low expected frequencies, Fisher’s exact test was considered the more appropriate reference for interpretation, and overall univariate analyses did not support statistically robust associations with treatment response, despite a consistent direction of effect across comparisons.

Analyses using remission status likewise did not identify significant categorical correlates.

### 3.3. Multivariable Analyses of Factors Associated with Clinical Response

To further examine factors associated with clinical response in this real-world cohort, exploratory parsimonious binary logistic regression models were performed with responder status as the dependent variable. In the main multivariable model, alcohol use disorder (AUD), cannabis use disorder (CUD), age, and sex were entered based on a priori clinical relevance and to reduce overfitting risk in the context of the limited sample size.

In this model, both AUD and CUD were significantly associated with lower odds of clinical response. Specifically, AUD showed reduced odds of response (OR = 0.079, 95% CI 0.010–0.595, *p* = 0.014), as was CUD (OR = 0.243, 95% CI 0.068–0.860, *p* = 0.028), whereas age (OR = 0.972, 95% CI 0.915–1.034, *p* = 0.371) and sex (OR = 2.776, 95% CI 0.619–12.443, *p* = 0.182) were not significantly associated with response (Table 3).

Sensitivity analyses yielded directionally consistent findings. In a model including ASD, both AUD (OR = 0.106, 95% CI 0.015–0.766, *p* = 0.026) and CUD (OR = 0.202, 95% CI 0.056–0.726, *p* = 0.014) remained significantly associated with reduced response, whereas ASD showed a trend toward lower response but did not retain a stable adjusted association (OR = 0.227, 95% CI 0.036–1.428, *p* = 0.114). Similarly, in a model including stimulant treatment exposure, AUD (OR = 0.074, 95% CI 0.010–0.537, *p* = 0.010) and CUD (OR = 0.210, 95% CI 0.059–0.745, *p* = 0.016) remained significantly associated with lower response, while stimulant exposure was not significant (OR = 0.572, 95% CI 0.157–2.087, *p* = 0.398).

When family history of neurodevelopmental disorders was examined in a separate sensitivity model, it showed a positive trend that did not reach conventional statistical significance (OR = 4.991, 95% CI 0.940–26.488, *p* = 0.059), while the associations for AUD (OR = 0.089, 95% CI 0.012–0.655, *p* = 0.017) and CUD (OR = 0.217, 95% CI 0.060–0.777, *p* = 0.019) remained broadly stable.

Across parsimonious and sensitivity models, AUD and CUD showed the most consistent exploratory associations with reduced clinical response, whereas findings related to ASD and family history of neurodevelopmental disorders were not sufficiently stable to support firm conclusions.

**Figure 1:**
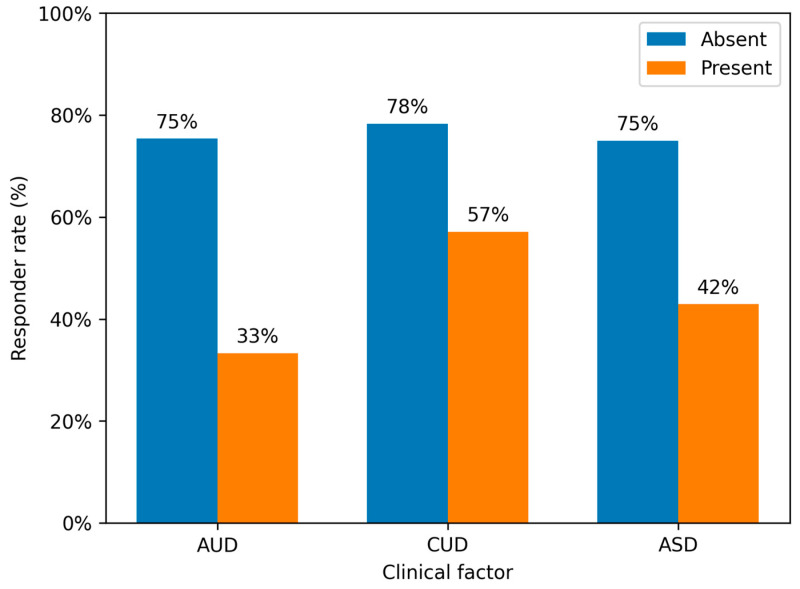
Responder rates according to alcohol use disorder (AUD), cannabis use disorder (CUD), and autism spectrum disorder (ASD). For each factor, the proportion of patients meeting criteria for clinical response (CGI-I ≤ 3) is shown separately for individuals with and without the condition. Lower responder rates were observed in patients with AUD (33.3% vs. 75.4%) and CUD (57.1% vs. 78.3%), while ASD was associated with an intermediate pattern (42.9% vs. 75.0%). These findings should be interpreted cautiously given the limited sample size and the exploratory nature of the analyses.

A graphical representation of responder rates across AUD, CUD, and ASD groups is shown in Figure 1.

## 4. Discussion

This exploratory retrospective study examined clinical and psychopathological correlates of pharmacological treatment response in adults with ADHD, with a specific focus on SUD. Within this naturalistic clinical cohort, AUD and CUD showed the most consistent exploratory associations with a lower likelihood of clinical response across parsimonious multivariable and sensitivity models.

Beyond substance-related factors, ASD showed a trend toward lower response rates, but findings were not stable enough to support firm conclusions across sensitivity analyses, likely reflecting limited statistical power given the small number of ASD cases, whereas a family history of neurodevelopmental disorders showed a positive trend that did not reach conventional statistical significance. These findings may suggest that treatment outcomes in adult ADHD reflect not only core symptom severity, but also broader neurodevelopmental profiles and substance-related vulnerabilities, although these associations should be interpreted cautiously given their limited stability.

Taken together, the results are broadly consistent with existing models that conceptualize adult ADHD as a markedly heterogeneous neurodevelopmental condition, in which variability in treatment response appears to reflect the interaction between developmental vulnerabilities and substance use trajectories rather than a uniform pharmacological effect [56,57]. Moreover, evidence indicates that ADHD developmental trajectories are strongly intertwined with substance-use risk across adulthood, supporting the relevance of considering substance-related trajectories when interpreting treatment variability in complex adult ADHD samples [11,58]. Although the retrospective observational design precludes causal inferences, the high rates of psychiatric comorbidity observed in the present cohort are consistent with those reported in real-world adult ADHD populations, supporting the ecological validity of these findings [38,41,59].

### 4.1. Alcohol and Cannabis Use Disorders as Clinical Correlates of Reduced ADHD Treatment Response

Although the present study’s retrospective design precludes causal inference, the consistency of associations observed across parsimonious multivariable models may suggest that alcohol and cannabis use represent clinically relevant correlates of reduced treatment response in adult ADHD. This interpretation is consistent with previous literature indicating that comorbid SUDs are linked to poorer functional outcomes, reduced treatment adherence, and greater clinical complexity in ADHD populations [60,61]. However, prior studies have often grouped SUDs together or focused primarily on stimulant use disorders, yielding heterogeneous and sometimes contradictory results [62]. The present study adds preliminary real-world evidence, in a hypothesis-generating framework, suggesting that alcohol and cannabis use disorders may be linked to a lower probability of achieving a clinically observable response, relative to other substance use categories examined in the present sample, although this finding should be interpreted cautiously given the exploratory nature of the analyses.

The current findings are largely consistent with the existing, albeit limited, available literature. Research from randomized controlled trials, systematic reviews, and meta-analyses indicates that ADHD medications maintain effectiveness in individuals with comorbid SUDs, though with reduced effect sizes compared to non-comorbid ADHD populations [60,63,64,65]. It is important to note that most of the available evidence derives from studies conducted in adolescents or mixed-age samples, and data specifically focusing on adults with distinct SUD subtypes, such as AUD or CUD, remain limited, constraining direct comparisons with real-world adult cohorts such as the present one.

Within this context, adult-specific evidence is relatively more developed for AUD. A placebo-controlled randomized trial of atomoxetine in adults with ADHD and comorbid AUD demonstrated improvements in ADHD symptoms alongside reductions in heavy drinking days [66]. However, treatment effects were more pronounced among individuals achieving partial abstinence, and overall effect sizes were lower than those reported in non-comorbid adult ADHD populations [67]. In contrast, evidence regarding CUD remains scarce, particularly in adults. Available observational data suggest that, although ADHD medications may improve core symptoms, ongoing cannabis use may be associated with less pronounced overall clinical improvement in some patients, and is not consistently reduced by pharmacological treatment [62,68,69,70].

Evidence reporting an association between co-occurring AUD and CUD and the probability of achieving a clinically meaningful response to ADHD pharmacotherapy should be interpreted cautiously and not considered conclusive. However, these findings are broadly consistent with a substantial body of literature identifying ADHD as a high-risk neurodevelopmental condition for the development of substance-related disorders across the lifespan [71,72,73]. This is especially the case when ADHD symptoms persist into adulthood and are accompanied by impairments in executive control and reward processing [12,74,75]. Moreover, the elevated prevalence of SUDs in adults with ADHD, markedly higher than in non-ADHD populations [76,77], has been interpreted as reflecting an increased vulnerability to developing addictive trajectories, characterized by impaired self-regulation, altered reward processing, and deficient inhibitory control [78,79,80,81]. From this perspective, the key clinical issue may relate not only to the risk of initial exposure, but also to the progression from use to misuse and addiction, potentially reflecting abnormalities in “top-down” executive control and reinforcement learning mechanisms that are central to both ADHD and addiction [82,83,84]. Consistently, longitudinal evidence indicates that hyperactivity–impulsivity traits are primarily associated with earlier initiation of alcohol use during adolescence, whereas the persistence of alcohol-related behaviors into adulthood appears to depend more strongly on subsequent neuroadaptive and environmental processes [85].

Within this framework, the specific association of alcohol and cannabis with poorer response to ADHD pharmacotherapy appears clinically plausible but remains inferential in the context of the present data and should not be interpreted as evidence of mechanism within the present dataset. Both substances have been repeatedly linked, through partially distinct mechanisms, to dysfunction in executive domains that are critical for ADHD outcomes, including attention regulation, working memory, inhibitory control, emotional regulation, and motivational processes [86,87,88,89]. Furthermore, both cannabis and alcohol have been linked to neuroadaptive changes, including upregulation of the DAT (dopamine transporter), a mechanism related to reduced dopamine signaling efficiency in the forebrain [24,90]. However, these neurobiological mechanisms were not directly assessed in the present study and should therefore be interpreted as hypothetical explanatory frameworks rather than demonstrated mechanisms.

Specifically, chronic alcohol use has been associated with abnormal reward processing and impaired emotional self-regulation through dysregulation of dopaminergic reward pathways and fronto-striatal dysfunction. Long-term alcohol exposure has been linked to alterations in dopamine release and dopamine receptor function in prefrontal and striatal regions, contributing to deficits in inhibitory control, cognitive flexibility, and decision-making-processes that underpin core executive functions. In addition, as a central nervous system depressant, alcohol may disrupt cortical inhibitory networks, further weakening top-down regulatory control and amplifying pre-existing impulse-control deficits [91]. These neuroadaptive changes have been associated with reduced sensitivity of the mesolimbic reward system to both substance-related and natural reinforcers and could theoretically interfere with the neurobiological mechanisms through which ADHD medications exert their effects [30,92,93].

In a similar manner, cannabis use has been associated with altered dopaminergic function, including reduced dopamine synthesis capacity in the striatum in chronic users as shown in molecular imaging studies of human cannabis users [22]. The main psychoactive component of cannabis, THC, interacts with the endocannabinoid system and has downstream effects on dopaminergic, glutamatergic and GABAergic signaling, with impacts on executive, emotional, reward, and memory processing [94]. Long-term regular cannabis use has been associated with altered activation of executive and default mode networks, particularly in attentional and control systems, even after periods of abstinence [95]. Additionally, chronic use has been associated with impaired working memory and implicit learning, likely reflecting disruptions in corticostriatal pathways that underlie complex cognitive and executive functions [96]. Cannabinoid modulation of mesocorticolimbic dopamine release has been proposed as a potential mechanism through which cannabis exposure may influence motivation, reward processing, and behavioral regulation [97]. While these findings provide biological plausibility, they cannot be directly extrapolated to the present sample.

Furthermore, neuroimaging studies have shown that heavy or prolonged cannabis use is associated with altered activation and connectivity in brain networks supporting executive and memory functions, while longitudinal evidence suggests associations between cannabis exposure and subsequent impairments in attentional and inhibitory control [95,98,99,100,101,102,103]. In parallel, epidemiological and clinical data consistently support a strong association between ADHD and later alcohol-related problems, underscoring the relevance of AUD within ADHD developmental trajectories [104,105,106,107,108,109,110].

Accordingly, a randomized controlled neuroimaging study reported that regular cannabis users showed diminished responses to MPH, including reduced changes in striatal distribution volumes and decreased brain reactivity to dopaminergic stimulation [24].

These findings have been interpreted by the authors as reflecting reduced responsivity of reward and emotional regulation circuits. Such alterations have been hypothesized to contribute to affective dysregulation, including irritability, stress vulnerability, and emotional instability, as well as to maladaptive reward-seeking behaviors. However, these interpretations are based on experimental data and may not directly generalize to clinical populations. Although informative, such findings derive from experimental settings and were not assessed in the present clinical cohort.

A clinically nuanced interpretation of these findings is that, in a subset of adults, ADHD may, in some cases, become less compensated or more clinically evident over time in the context of chronic substance exposure. Sustained alcohol or cannabis use may be associated with neuroadaptive changes and potential neurotoxic effects that could further impair executive functioning and amplify emotional dysregulation, potentially contributing to a reduced likelihood of achieving optimal clinical response to ADHD-targeted pharmacotherapy in routine clinical practice [111,112,113,114,115,116,117]. This interpretation remains speculative and should be regarded as hypothesis-generating. It does not imply that alcohol or cannabis cause ADHD in a straightforward manner. Rather, it supports a cautious and hypothesis-generating model in which substance-induced executive deterioration may unmask or exacerbate pre-existing neurodevelopmental vulnerabilities, leading to clinical presentations that may overlap phenomenologically with ADHD, while potentially differing in underlying pathophysiology and, consequently, in treatment responsiveness. Given the retrospective design of the present study, this interpretation must be regarded as exploratory. Importantly, the present findings do not imply that ADHD pharmacotherapy is ineffective in patients with AUD or CUD, but rather that the likelihood of achieving a global, clinically observable improvement is reduced. This distinction has practical implications, underscoring the need for integrated treatment approaches that systematically address substance use alongside ADHD pharmacotherapy.

Nevertheless, the attenuated clinical response observed in the present study may also be explained by other relevant factors. First, reduced adherence to prescribed treatment regimens may have contributed to the observed outcomes. Adults with ADHD have been consistently shown to exhibit lower levels of medication adherence, largely due to core symptoms such as difficulties with organization, planning, and prospective memory [118]. In addition, individuals with comorbid ADHD and SUDs report greater challenges in maintaining treatment adherence compared to patients with either condition alone, reflecting the added burden of substance-related behaviors and psychosocial instability [62]. As treatment adherence was not systematically assessed in the present study, its potential impact should be considered when interpreting the findings.

Second, suboptimal pharmacological dosing may have played a role in the reduced treatment response observed in this clinically complex population. Evidence from real-world studies and clinical trials suggests that standard stimulant dosing regimens may not always be sufficient for some adults with ADHD, particularly in the presence of comorbid conditions, and that careful dose optimization, sometimes involving higher doses within or near the upper recommended range (e.g., up to approximately 90 mg/day of methylphenidate), may be required to achieve meaningful clinical improvement [60,61,62,119,120,121]. In this context, conservative dosing strategies in patients with comorbid substance use disorders may inadvertently limit treatment effectiveness. Given the lack of standardized data on dosage and cumulative exposure, this represents a relevant potential confounding factor in the present study.

### 4.2. Other Substance Use Disorders and Clinical Heterogeneity in ADHD–SUD Comorbidity

Notably, other SUD categories, including stimulant use disorder, were not retained as stable adjusted correlates in this sample of treatment response in the final model. This finding should be interpreted cautiously, given the limited sample size and the low prevalence of several SUD subtypes. Rather than indicating a true absence of effect, this result likely reflects insufficient statistical power to detect substance-specific associations beyond alcohol and cannabis.

However, it is also possible that stimulant use disorder interacts more strongly with treatment selection (e.g., preferential use of non-stimulant medications) than with treatment response per se, an aspect that could not be formally tested in the present study due to sample size constraints. More broadly, differences across substance use profiles may reflect underlying clinical heterogeneity rather than distinct mechanistic pathways directly demonstrated in the present data.

Some clinically oriented hypotheses have suggested that individuals with a more “core” ADHD neurobiology may be more likely to engage in stimulant use, potentially as a form of self-medication [122,123,124,125], whereas other profiles characterized by marked behavioral disinhibition and heightened reward sensitivity (“hyper-impulsive” profile) may be more frequently associated with alcohol use. However, these interpretations remain speculative and cannot be directly tested within the present dataset [31,126,127].

By comparison, within the context of this topic, chronic non-stimulant use has been found to be more closely linked to a broader psychopathological ADHD symptomatology, such as emotional self-regulation strategies, sleep disturbances, and cumulative executive inefficiency, than to a core ADHD endophenotype. This phenomenon is particularly salient among chronic cannabis users [64,99,128]. Notably, these clinical phenomena typically fall outside the diagnostic criteria for core ADHD [20,129].

Taken together, these observations may suggest the presence of partially distinct clinical patterns; however, this cannot be directly tested within the present study. Therefore, while ADHD is widely recognized as a robust transdiagnostic risk factor for SUDs across substances, epidemiological and clinical evidence suggests that alcohol, cannabis, stimulant, and opioid use disorders interact with ADHD in partially distinct ways, with specific substances of abuse contributing to meaningful differences in demographic characteristics, associated psychopathology, and illness trajectories.

This perspective supports the view that ADHD–SUD comorbidity is not homogeneous, although the extent to which these differences translate into differential treatment response remains to be clarified. Accordingly, the present findings should be interpreted as exploratory and hypothesis-generating, rather than as evidence of substance-specific effects on pharmacological outcomes [130,131,132].

Specifically, meta-analyses and large observational studies consistently report a markedly elevated prevalence of ADHD across SUD populations, ranging from approximately 18–25% in alcohol, opioid, and stimulant use disorders, with comparable or slightly higher estimates in cannabis-using samples [12,133]. However, the preponderance of extant evidence stems from adolescent or mixed-age cohorts, with adult-only data remaining comparatively scarce. Nonetheless, substance-specific demographic patterns have been delineated. For example, CUD appears to be particularly prevalent among adults diagnosed with ADHD. Research studies have reported earlier onset, longer duration of use, and a relatively higher representation of females compared with other SUD subtypes [76,85,134,135]. In contrast, AUD and stimulant use disorders are more frequently observed in males and are often associated with early externalizing trajectories and impulsive behavioral profiles [65,136,137]. OUD, a condition that has received comparatively less research attention, has been found to exhibit a persistently high prevalence of ADHD in treatment-seeking populations, frequently in the context of multifaceted and clinical complexities [40,138,139]. From a clinical perspective, individuals with ADHD who also meet criteria for SUD tend to show greater overall severity, functional impairment, and psychiatric comorbidity than those without SUD [77,140,141]. However, qualitative differences in clinical presentation appear to depend on the substance involved. According to the extant literature, the use of stimulants and alcohol in individuals diagnosed with ADHD is more often associated with prominent hyperactivity–impulsivity, behavioral disinhibition, and legal or occupational problems [16,132]. Conversely, the use of cannabis is more frequently linked to broader psychopathological features, including affective instability, sleep disturbances, and executive inefficiency [64,128,142,143,144].

A notable finding is that adult studies comparing substance-specific groups generally do not find substantial differences in baseline core ADHD symptom severity. This observation has been interpreted as suggesting that substances may differentially modulate associated clinical features rather than the core ADHD phenotype itself, although this interpretation remains indirect [132]. Furthermore, impulsivity has been identified as a transdiagnostic dimension among all SUD subtypes, though its expression varies. AUD is more closely associated with emotional dysregulation and negative urgency, whereas OUD and stimulant use disorders are linked to sensation seeking and risk-taking behaviors [40,66]. Also, the presence of concomitant psychiatric disorders, including bipolar disorder, conduct disorder, antisocial personality disorder, or eating disorders, has been demonstrated to be associated with an elevated probability of concurrent AUD [145]. Furthermore, adolescents with comorbid ADHD and conduct disorder demonstrate an increased propensity for tobacco and alcohol use [146].

With respect to treatment, the substance class influences medication choice more than treatment response. According to clinical guidelines and reviews, the use of stimulants should be done with caution in individuals with stimulant use disorder. These reviews often favor non-stimulant options such as atomoxetine due to safety considerations rather than evidence of reduced efficacy [49,60,62,147]. Across substances, systematic reviews indicate that ADHD medications remain effective in reducing ADHD symptoms, though with attenuated effect sizes compared to non-comorbid ADHD populations, and without consistent evidence of differential efficacy by substance type [64]. Likewise, the long-term outlook is consistently more unfavorable in cases involving any SUD, marked by earlier onset, higher rates of relapse, and more significant psychosocial impairment. While specific risks vary, such as higher injury and mood disorder burden in AUD, or elevated medical and legal morbidity in stimulant and opioid use, current evidence does not support distinct prognostic models based solely on substance class [76,93]. These epidemiological and clinical differences support the view that ADHD–SUD comorbidity is heterogeneous, rather than reflecting a single uniform developmental pathway. However, the extent to which substance-specific differences translate into differential treatment response remains uncertain.

In this context, the findings of the present study should be interpreted cautiously. While alcohol and cannabis use disorders were associated with reduced odds of clinical response in multivariable models, these results do not demonstrate substance-specific causal effects and may reflect broader clinical complexity, residual confounding, or unmeasured factors such as treatment adherence, pharmacological exposure, and severity of substance use.

Within a broader theoretical framework, these observations may be considered in light of integrative models suggesting dynamic interactions between ADHD, substance use, and neuroprogressive processes across development and adulthood; however, such mechanisms were not directly assessed in the present study and remain inferential [148,149,150]. Accordingly, although some theoretical models have proposed that distinct substance use patterns may be associated with different neurobehavioral profiles, the present data do not allow the identification of distinct neurobiological subtypes or “endophenotypes”.

From a clinical perspective, these findings underscore the importance of systematically assessing substance use in adults with ADHD, while avoiding overinterpretation of substance-specific effects. Alcohol and cannabis use may represent potential clinical markers of greater clinical complexity rather than direct explanations for reduced treatment response.

Failure to identify and address these factors may contribute to suboptimal treatment outcomes in some patients; however, further prospective studies with larger samples and detailed characterization of treatment exposure and substance use patterns are required to clarify these relationships.

### 4.3. ASD and Family History of Neurodevelopmental Disorders in Relation to Treatment Response

In addition to substance-related factors, ASD showed a trend toward lower odds of clinical response in some exploratory multivariable models, whereas a family history of neurodevelopmental disorders showed a positive trend that did not reach robust statistical significance across sensitivity analyses. Given the limited sample size, the variability across models, and the small number of ASD cases, these findings should be interpreted with caution.

Rather than supporting the presence of distinct neurodevelopmental phenotypes with clearly different treatment responsiveness, these results may reflect broader clinical heterogeneity within adult ADHD populations. The trend toward lower response observed in individuals with co-occurring ASD is broadly consistent with some evidence suggesting that ADHD–ASD comorbidity may be characterized by greater cognitive rigidity, atypical executive organization, and altered reward processing, which could contribute to less favorable outcomes under standard ADHD pharmacotherapies [9,151]. However, these mechanisms were not directly assessed in the present study and the observed association was not stable enough to support firm conclusions.

At the same time, it cannot be excluded that, in some cases, this clinical presentation reflects overlapping or partially distinct neurodevelopmental profiles, rather than a simple additive comorbidity of two separate disorders. In this context, global improvement as measured by the CGI-I may be more difficult to achieve, even in the presence of partial symptom reduction, potentially contributing to the observed variability in treatment response. Accordingly, these findings highlight the importance of considering outcome measures and clinical expectations in patients with co-occurring ADHD and ASD, while avoiding overinterpretation of subgroup-specific effects.

An intriguing finding is the association between a family history of neurodevelopmental disorders and a higher likelihood of treatment response. However, this effect was characterized by wide confidence intervals and limited precision, and should therefore be considered preliminary. Possible interpretations include greater diagnostic clarity, earlier recognition of symptoms, or higher levels of psychoeducation and engagement with treatment. Alternatively, family history may identify a subgroup with a more “prototypical” presentation of ADHD, potentially less influenced by secondary psychopathology or substance-related factors. These interpretations remain speculative and cannot be confirmed within the present dataset, although they are broadly consistent with developmental models emphasizing the role of genetic loading and neurobiological continuity in shaping adult ADHD outcomes [152]. Overall, findings related to ASD and family history should be regarded as exploratory and in need of replication in larger samples with more detailed characterization of neurodevelopmental features and treatment exposure.

### 4.4. Limitations

Several limitations of the present analysis should be acknowledged. First, the retrospective and naturalistic design precludes causal inference and relies on the accuracy and completeness of routinely collected clinical data. In addition, the absence of randomization and standardized treatment allocation reflects routine clinical practice but limits control over potential confounding by indication. Although this approach enhances ecological validity, it inherently reduces control over confounding variables and the temporal precision of clinical assessments.

Second, the sample size, particularly within specific comorbidity subgroups (i.e., alcohol use subgroup), was relatively limited, and no a priori sample size calculation was performed, which may have reduced statistical power to detect smaller effects and may partially account for the lack of stable adjusted associations observed for some clinical variables and SUD categories. The relatively small number of non-responders further limited the stability of multivariable estimates.

Third, SUDs were operationalized as dichotomous variables, without detailed characterization of severity, duration, patterns of use, or temporal proximity to ADHD treatment initiation. Although SUD cases in the present study reflected clinically assessed active substance use disorders at baseline, the dataset did not include a standardized dimensional characterization of substance use (e.g., severity, frequency, duration, or time since onset), nor longitudinal information on changes in substance use during follow-up. This simplification, while necessary given the retrospective nature of the data, may have obscured dose-dependent or stage-specific effects of substance use on treatment response and does not fully capture the dimensional nature of addiction and neurodevelopmental conditions. Accordingly, the observed associations should be interpreted as reflecting the presence of an active SUD diagnosis at baseline rather than specific substance use trajectories or exposure intensity.

Fourth, the outcome definition warrants consideration. The exclusive use of the CGI-I introduces a subjective component and does not allow for the distinction between specific improvements in ADHD symptoms and non-specific overall improvements, as there is a lack of sensitivity analyses based on dimensional changes (ASRS, CAARS), and the dichotomization of response may have further reduced sensitivity to gradations of clinical change.

Fifthly, univariate analyses are largely insignificant, whereas marked effects emerge in the multivariate model, suggesting possible suppression or model-dependent effects. Although this discrepancy may reflect adjustment for confounding and intercorrelations among variables, model instability cannot be excluded given the limited sample size, and therefore the findings should be interpreted cautiously. Accordingly, the multivariable results should be considered exploratory and hypothesis-generating rather than confirmatory.

Finally, the heterogeneity of pharmacological treatments reflects real-world practice but also introduces additional variability that may have influenced outcomes. Moreover, detailed and standardized data on medication adherence, dosage optimization, and cumulative pharmacological exposure were not consistently available and were not included as quantitative variables, which represents a relevant limitation. Therefore, differences in treatment response may be partially influenced by variability in actual drug exposure rather than solely by patient-related clinical characteristics.

Future prospective studies with standardized treatment protocols, systematic assessment of adherence and pharmacological exposure, longitudinal substance use assessment, and multimodal outcome measures are warranted to further clarify the observed associations.

## 5. Conclusions

In this retrospective real-world study of adults with ADHD, alcohol and cannabis use disorders were associated with lower odds of clinical response in parsimonious multivariable models, whereas ASD showed a trend toward lower response that was not stable across sensitivity analyses, and a family history of neurodevelopmental disorders showed a positive trend that did not reach conventional statistical significance. Given the limited sample size and observational design, these findings should be interpreted as exploratory rather than definitive. By focusing on a real-world adult cohort and on clinician-rated global improvement under routine pharmacological care, the present study contributes preliminary evidence suggesting that specific SUD subtypes, particularly alcohol and cannabis use disorders, may contribute to variability in overall clinical response beyond what is captured by symptom-specific outcomes.

Beyond their role as comorbid diagnoses, alcohol and cannabis use disorders may represent clinical markers associated with a lower probability of achieving a clinically observable improvement under routine treatment conditions, rather than established causes of reduced pharmacological response. The present data do not allow causal inference, and alternative explanations, including residual confounding, variability in treatment adherence, and differences in pharmacological exposure, cannot be excluded.

Although neurobiological models have proposed mechanisms linking substance use to executive dysfunction and altered treatment responsiveness, these mechanisms were not directly assessed in the present study and should therefore be considered speculative in this context.

From a clinical perspective, these results highlight the need for an integrated and comprehensive approach to adult ADHD, in which alcohol and cannabis use are systematically assessed as part of routine clinical evaluation. However, these factors should be interpreted as indicators of increased clinical complexity rather than as established causes of reduced treatment response. Future longitudinal and prospective studies are warranted to clarify the temporal and mechanistic relationships between substance use, treatment exposure, and ADHD outcomes.

In summary, this real-world study indicates that alcohol and cannabis use disorders may be associated with a reduced likelihood of achieving clinically meaningful improvement following pharmacological treatment for adult ADHD, although these findings require replication. These findings support the inclusion of substance use assessment in routine ADHD care, while avoiding overinterpretation of substance-specific effects.

Despite these limitations, the present findings have potential clinical implications. They suggest that systematic assessment and active management of alcohol and cannabis use should be considered components of adult ADHD care. At the same time, further research is needed to determine whether targeted interventions for substance use can improve treatment outcomes in this population.

Future investigations should include larger samples, prospective designs, dimensional measures of substance use and neurodevelopmental features, and detailed characterization of treatment exposure, including adherence and dosage. Such approaches will be essential to better characterize substance use as a marker of clinical complexity and to clarify its relationship with overall treatment response in adult ADHD under real-world conditions.

## Figures and Tables

**Table 1:** Demographic and clinical characteristics of the sample (N = 67).

Variable	n (%) or Mean ± SD
**Demographics**	
Age (years)	29.09 ± 10.20
Female sex	21 (31.3%)
**ADHD Presentation**	
ADHD combined	45 (67.2%)
ADHD inattentive	17 (25.4%)
ADHD hyperactive/impulsive	5 (7.5%)
**Psychiatric Comorbidities**	
Mood disorders	42 (62.7%)
Anxiety disorders	36 (53.7%)
Personality disorders	20 (29.9%)
Eating disorders	3 (4.5%)
**Substance Use Disorders**	
Any SUD	37 (55.2%)
Cannabis use disorder	21 (31.3%)
Stimulant use disorder	9 (13.4%)
Alcohol use disorder	6 (9.0%)
Opioid use disorder	1 (1.5%)
**Neurodevelopmental Profile**	
Specific learning disorder	16 (23.9%)
ASD	7 (10.4%)
Tic disorder	6 (9.0%)
Intellectual disability	6 (9.0%)
**Pharmacotherapy**	
Methylphenidate	45 (67.2%)
Atomoxetine	17 (25.4%)
Bupropion	5 (7.5%)
**Clinical Outcomes**	
Responders	48 (71.6%)
Remission	5 (7.5%)

Note. Bold indicates main category headings.

**Table 2:** Clinical response according to AUD, CUD, and ASD.

Variable	Responders n (%)	Non-Responders n (%)	*p*-Value
Alcohol Use Disorder			
Present	2 (33.3%)	4 (66.7%)	0.050 *
Absent	46 (75.4%)	15 (24.6%)	
Cannabis Use Disorder			
Present	12 (57.1%)	9 (42.9%)	0.088 *
Absent	36 (78.3%)	10 (21.7%)	
Autism Spectrum Disorder			
Present	3 (42.9%)	4 (57.1%)	0.094 *
Absent	45 (75.0%)	15 (25.0%)	

* Fisher’s exact test was used due to low expected cell counts.

**Table 3:** Parsimonious multivariable logistic regression models of factors associated with clinical response.

Variable	B	SE	Wald	*p*	OR	95% CI
AUD	−2.543	1.033	6.065	0.014	0.079	0.010–0.595
CUD	−1.416	0.646	4.812	0.028	0.243	0.068–0.860
Age	−0.028	0.031	0.800	0.371	0.972	0.915–1.034
Sex	1.021	0.765	1.780	0.182	2.776	0.619–12.443

## Data Availability

The data supporting the findings of this study are not publicly available due to privacy and ethical restrictions, as they contain sensitive clinical information. De-identified data may be made available from the corresponding author upon reasonable request and subject to institutional and regulatory approval.

## Abbreviations

The following abbreviations are used in this manuscript:

ADHD	Attention-Deficit/Hyperactivity Disorder
ASD	Autism Spectrum Disorder
ASRS	Adult ADHD Self-Report Scale
ATX	Atomoxetine
AUD	Alcohol Use Disorder
BPRS-6	Six-item Brief Psychiatric Rating Scale
BRI	Behavioral Regulation Index
BRIEF-A	Behavior Rating Inventory of Executive Function—Adult Version
CAARS-O:SV	Conners’ Adult ADHD Rating Scale—Observer Report: Screening Version
CGI-I	Clinical Global Impression—Improvement scale
CI	Confidence Interval
CUD	Cannabis Use Disorder
DAT	Dopamine Transporter
DIVA-5	Diagnostic Interview for ADHD in Adults, version 5
DSM-5-TR	Diagnostic and Statistical Manual of Mental Disorders, Fifth Edition, Text Revision
DSM-5	Diagnostic and Statistical Manual of Mental Disorders, Fifth Edition
DSP	Delayed Sleep Phase profile
HAM-D	Hamilton Depression Rating Scale
HED	Higher Emotional Dysregulation
LED	Lower Emotional Dysregulation
MEQ	Morningness–Eveningness Questionnaire
MI	Metacognition Index
MPH	Methylphenidate
OR	Odds Ratio
OUD	Opioid Use Disorder
RIPoSt-40	Reactivity, Intensity, Polarity and Stability questionnaire
SCID-5	Structured Clinical Interview for DSM-5
SUD	Substance Use Disorder
THC	Δ9-tetrahydrocannabinol
YMRS	Young Mania Rating Scale
